# Patient-centered pregnancy planning in multiple sclerosis: evidence for a new era

**DOI:** 10.1055/s-0044-1791202

**Published:** 2024-10-02

**Authors:** Elisa Matias Vieira de Melo, Bruno Cassis Antunes Rodrigues, Felipe Teijeiro Cabral, Luíza Alves Monteiro Torreão Villarim, Maria Fernanda Mendes

**Affiliations:** 1 Santa Casa de São Paulo, Faculdade de Ciências Médicas, Departamento de Neurologia, São Paulo SP, Brazil.

**Keywords:** Multiple Sclerosis, Pregnancy, Postpartum Period, Breastfeeding, Family Development Planning, Esclerose Múltipla, Gravidez, Período Pós-Parto, Aleitamento Materno, Planejamento Familiar

## Abstract

A few decades ago, women diagnosed with multiple sclerosis were discouraged from becoming pregnant. However, with new knowledge about the disease and treatments, this recommendation has changed, and it is pregnancy after the diagnosis of the disease is no longer contraindicated, with family planning being essential in this process. This review aims to provide a comprehensive overview of the family planning process for people with multiple sclerosis.

## Resumo

Até recentemente, a gravidez nas pacientes com o diagnóstico de esclerose múltipla era contraindicada. O avanço no conhecimento sobre a doença e os tratamentos alterou essa recomendação, e agora a gravidez após o diagnóstico da doença não é mais contraindicada; contudo, o planeamento familiar é essencial nesse processo. Esta revisão tem como objetivo fornecer uma visão abrangente do processo de planejamento familiar para pacientes com esclerose múltipla.

## INTRODUCTION

The gender-based prevalence ratio of multiple sclerosis (MS) is 3:1, indicating a higher prevalence among women compared with men, as is the case with most autoimmune diseases. 1 In Brazil, the prevalence varies from 5 to 20 individuals per 100,000 inhabitants, 2 predominantly, as in the rest of the world, in women of reproductive age. A retrospective analysis conducted using the public health registry between 2000 and 2015 found a total of 28,401 MS patients being treated in the country, with an average age of 36.8 years, with the majority being female (73.3%) and residing in the southeastern region of Brazil (58.9%). 3

In the past, women diagnosed with MS were discouraged from becoming pregnant due to fear of being unable to care for their children due to fatigue or disability, concerns about transmitting genetic susceptibility to an autoimmune condition to their children, as well as about the use of disease-modifying therapies (DMTs) required to control disease activity. 4 However, accumulated scientific knowledge about the disease, and attitudes toward illness have changed, meaning that health professionals can now routinely discuss family planning issues with patients and plan pregnancy while aiming to ensure both the safety of the patient (by maintaining disease control measures) and the baby (avoiding teratogenicity and negative outcomes during pregnancy, childbirth, and postpartum). This means that, if it is the patient's wish, this desire to become pregnant can now be realized. This review aims to provide a comprehensive overview of the family planning process for people with MS, covering all aspects of pregnancy (fertility, supplementation, examinations, the role of the multidisciplinary team) as well as the use of medications during pregnancy and breastfeeding.

## FERTILITY

There is no data to suggest that MS alters the fertility of patients, 5 but there are factors indirectly related to the desire to conceive that can interfere with fertility. These include bladder dysfunction, sexual dysfunction, pain, and mood disorders, particularly depression, and they should be addressed as confounding factors in infertile couples. 6 In a recent study, our group observed the widespread use of low-effectiveness contraceptive methods, such as the pill, patch, vaginal ring, male condom, and female condom (68.7%), and found that 70.1% of the patients in the same study do not plan to become pregnant within the next 2 years. 7 These data are consistent with those of López-Reyes et al., and highlight the importance of counseling on contraception with high-effectiveness methods, such as the intrauterine device (IUD), vasectomy, and tubal ligation, as the use of low-efficacy contraceptive methods may result in unplanned pregnancies. 8

It is advisable for women with MS to wait for a period of disease stability before attempting to conceive, usually 1 to 2 years. As a result, they often postpone their desire for motherhood and may, therefore, be older than their healthy counterparts when they begin their pregnancy attempts, making them more likely to require assisted reproductive techniques than the general population, 9 even though they are less encouraged to do so than the general population. 10

Historically, hormonal treatment for assisted reproductive techniques has increased the risk of relapse, with the appearance or exacerbation of lesions on magnetic resonance imaging (MRI), particularly in the first 3 months after failure to conceive, and with the use of gonadotropin-releasing hormone (GnRH) agonists. 11 However, recent studies have indicated that there is no increase in relapses following the use of these techniques, probably due to changes in protocols, with shorter treatment durations, frequent use of GnRH antagonists, and the use of DMTs during fertility treatments. 10

Oocyte and embryo cryopreservation are alternative procedures to consider in cases of young women recently diagnosed with MS who will have to postpone their desire for motherhood. 12

## THE ACTIVITY OF MS DURING PREGNANCY AND BREASTFEEDING – CONSIDERATIONS ON PERFORMING MRI, PULSE THERAPY, AND PLASMAPHERESIS

### The activity of MS during pregnancy

Pregnancy has a protective effect on the course of MS, with reduced relapse rates due to the immunologically accommodating gestational state. However, there is an increased incidence of relapses in the postpartum period. 13 In this context, it is important to remember the Pregnancy in Multiple Sclerosis (PRIMS) study, the first study analyzing pregnancy and MS, which showed that the annualized relapse rate compared with the 12 months before pregnancy dropped from 0.7 to 0.2 in the third trimester of pregnancy, but increased to 1.2 in the first 3 months postpartum, with approximately 1/3 of women experiencing new relapses. 14

In modern cohorts, risk predictors for relapse include disease activity in the last year, patients under the age of 35, and discontinuation of DMTs, especially natalizumab and fingolimod, with a high risk of severe relapses (∼ 10%). Postpartum disease activity has been associated with disease activity and severity before pregnancy, relapses during pregnancy, and withdrawal of high-efficiency therapies before conception. 13 15 On the other hand, the use of natalizumab during pregnancy reduces the risk of relapse by 24% compared with discontinuation. 14 Although still understudied, disease activity after spontaneous or elective interrupted pregnancies and stillbirths is ∼ 10%. 6

### Diagnostic procedures – MRI and contrast

There are considerations to contraindicate the performance of MRI, especially during the first trimester, due to potential risk to the fetus, although there is evidence suggesting no increased risk of stillbirth, neonatal death, or congenital anomalies. Therefore, magnetic resonance imaging can be performed if it is necessary.

The use of gadolinium during pregnancy is contraindicated, as it is associated with an increased risk of stillbirth, neonatal death, and the development of other autoimmune diseases (intestinal, rheumatological, and dermatological) as it is excreted into the amniotic fluid. In the breastfeeding population, there is no contraindication to the use of gadolinium, as less than 0.04% of the administered gadolinium passes into the breast milk, making it safe during lactation. However, some healthcare professionals prefer to recommend waiting at least 24 hours after the examination to resume breastfeeding. In this situation, it is important to advise patients who will undergo the procedure to store breast milk in advance, as well as to express and discard breast milk for up to 24 hours after the procedure. 16

### Treatment of relapses during pregnancy

Corticosteroid therapy carries ambiguous risks of cleft palate, low birth weight, and neuronal development impairment. These effects have been more extensively studied with fluorinated corticoids. 17 Therefore, although controversial, corticosteroids should be avoided, especially during the first trimester of pregnancy. 18 In cases of non-severe symptoms, such as mild sensory symptoms without significant functional impairment, it is advisable to consider the possibility of not using corticosteroid treatment. 6

Prednisone and methylprednisolone have minimal passage through the placental barrier and a short half-life. In practice, for relapses during the second and third trimesters, in the presence of disabling symptoms, considering the risk-benefit, corticosteroid treatment is recommended, with 1 g of intravenous methylprednisolone daily for 3 to 5 days. 18 19

Although intravenous immunoglobulin (IVIG) can be safely used during pregnancy and postpartum, it is not effective in the treatment or prevention of relapses during pregnancy and the postpartum period. 20 21

The use of therapeutic plasmapheresis in pregnant patients with MS is under-researched but may be beneficial as an alternative to corticosteroids in the first trimester of pregnancy and in cases of relapse of MS refractory to corticosteroids. 22 23

### Relapses during breastfeeding

Treatment with intravenous methylprednisolone has a relative infant dose (RID) of ∼ 1.5%, and it is advisable to wait for 2 to 4 hours after infusion before resuming breastfeeding to reduce the concentration of the medication in breast milk. 24 For plasmapheresis, data are limited, but no adverse effects have been reported. It should be considered on a case-by-case. 25 26

## MODES OF DELIVERY

Although women with MS have higher rates of labor induction and elective cesarean section, 27 there is no evidence of an increased risk of other adverse events or specific guidance on the delivery route. The choice of modes of delivery should follow obstetric indications. 28 Nevertheless, it is important to consider that these patients may have disabilities that could complicate delivery in certain obstetric procedures. For example, motor or sensory deficits in the lower limbs, ataxia, or cognitive deficits should be taken into account. Additionally, fatigue can be a limiting factor in prolonged labor. 29

## CONTRACEPTION

The most effective methods are long-acting, patient-independent reversible methods, such as intrauterine devices (IUDs) and implants. When considering the prescription of oral hormonal contraceptives, the following situations should be taken into account:

- Combined hormonal contraceptives may increase the risk of venous thromboembolism, which is further increased in patients with reduced mobility, such as those confined to a wheelchair. 30
- The reduced absorption of oral contraceptives due to diarrhea associated with dimethyl fumarate and the accelerated elimination protocol of teriflunomide.
- Interaction between oral contraceptives and antiepileptic drugs used for pain or paroxysmal symptoms. 6

## SUPPLEMENTATION

In patients with a desire for pregnancy, supplementation of folic acid, vitamin D, and iron should be performed, as is currently recommended for all women planning to conceive, according to local guidelines, as follows:

- Folic acid: Start supplementation 3 months before the beginning of the conception attempt.
- Vitamin D: Adjust supplementation to a maximum of 4,000 IU per day during pregnancy.
- Iron: Adjust replacement to prevent anemia before or during pregnancy. 6

## VACCINATION

Pregnant women are considered a priority vaccination group as they become susceptible to infections that can increase both mortality and morbidity. In general, vaccines containing live attenuated viruses are contraindicated during pregnancy, while inactivated vaccines are considered safe.

The general recommendation is to vaccinate against influenza and pertussis (tetanus, diphtheria, and acellular pertussis [Tdap] vaccines). Other vaccinations should be assessed on a case-by-case basis, considering vaccination updates, epidemics/pandemics, and negative serology for specific viruses (such as that of hepatitis B).

Vaccine titers should be confirmed before anti-CD20 or S1P receptor modulator treatments, as these medications can reduce the vaccine response. Furthermore, newborns who were exposed to anti-CD20 therapies during or shortly before pregnancy should be evaluated for CD19 levels before considering vaccination with live attenuated virus vaccines. 31

During breastfeeding, vaccines are generally considered safe, except for the yellow fever vaccine. 31 Coronavirus disease 2019 (COVID-19) vaccination can be administered during pregnancy and breastfeeding. 32

## PRENATAL CARE FOR MS PATIENTS

In comparison to healthy women, MS does not place patients at a higher risk of pregnancy complications, such as ectopic pregnancy, placental anomalies, spontaneous abortions, prepartum hemorrhages, preeclampsia, fetal demise, preterm birth, or congenital malformations. 33 34 35 Therefore, there is no recommendation for high-risk prenatal care based only on the diagnosis of MS.

## GENETIC RISK FOR DESCENDANTS OF MS PATIENTS

The risk varies between 2 and 3.5% and can reach 20% if both parents are affected. Although it represents a higher risk than that of the general population, it is much lower than what would be expected for a monogenic Mendelian disorder. Therefore, parents should be reassured that the risk of their children developing MS is low. 36 Consequently, there is no specific genetic counseling recommendation to assess the risk of MS.

## DISEASE MODIFYING THERAPIES DURING PREGNANCY AND BREASTFEEDING

### Pharmacokinetics during pregnancy

Small molecules (oral medications) cross the placental barrier, while large molecules, such as injectables, do not traverse it. Monoclonal antibodies have placental transport dependent on maternal plasma levels, the IgG subclass, and gestational age. For instance, in general, monoclonal antibodies can cross the placental barrier from the second trimester of pregnancy. 37 The recommended washout period varies for each medication, generally being advised as five half-lives. 6 For medications like fingolimod and natalizumab, there is a risk of rebound and relapse upon withdrawal. 38 Careful consideration is required when contemplating changes and discontinuation of these medications.

### Breastfeeding – general considerations

The excretion of medication in breast milk depends on factors such as molecular weight, bioavailability, and lactogenesis stage. For study purposes, the concept of RID is considered, which is the drug's oral bioavailability after excretion in breast milk. If the RID is above 10%, it is generally not tolerated.

Colostrum, produced in the first days after childbirth, has a different composition than that of mature breast milk. As most breastfeeding data primarily pertain to more than 14 days postpartum, it is recommended that most breastfeeding mothers wait until after this period to initiate MS treatment. Premature infants may experience higher drug absorption and poorer drug excretion than full-term infants, suggesting that some may need to wait more than 14 days. 39

## CONSIDERATIONS ON DMTs: PRECONCEPTION, PREGNANCY, AND BREASTFEEDING

The following will be considered regarding DMTs in relation to use before and during pregnancy, as well as during breastfeeding ( Table 1 ).

**Table 1:** Use of disease-modifying therapies (DMTs) during pregnancy and breastfeeding

DMT	Safe during pregnancy	Safe during breastfeeding
Interferon	Yes	Yes
Glatiramer	Yes	Yes
Teriflunomide	No	No
Dimethyl fumarate	At the moment, no. More evidence is needed for the approval of use during pregnancy	At the moment, no. More evidence is needed for the approval of use during breastfeeding
Fingolimod	No	No
Cladribine	No	No. Breastfeeding can be resumed after 7–10 days from the last ingested tablet
Natalizumab	Evaluate risk/benefit, it can be used during pregnancy with specific guidance	Yes
Ocrelizumab	Evaluate risk/benefit, it can be used during pregnancy with specific guidance	Yes
Ofatumumab	At the moment, no. More evidence is needed for the approval of use during pregnancy	Yes
Alemtuzumab	No	No. Breastfeeding can be resumed after 4 months from the last infusion day

### Interferon betas

#### *Preconception*

Regarding the safety of interferon use during pregnancy, there is no need for changes in the usual doses and intervals.

#### *Pregnancy*

It is recommended to use medications at the usual doses and intervals during pregnancy. For flu-like symptoms, paracetamol can be used. Avoid the use of ibuprofen after 28 weeks due to the risk of premature closure of the ductus arteriosus.

#### *Breastfeeding*

For interferon β1A, there is no minimum interval between drug administration and breastfeeding. The RID was estimated at ∼ 0.006%. No side effects or anomalies in infants were reported (n < 100). For flu-like symptoms, analgesic medications can be used. 6 40 41

### Glatiramer

#### *Preconception*

Regarding the safety of glatiramer use during pregnancy, there is no need for changes in the usual doses and intervals. 42

#### *Pregnancy*

It is recommended to use medications at the usual doses and intervals during pregnancy. 43

#### *Breastfeeding*

There is no minimum interval between drug administration and breastfeeding. Due to its higher molecular weight, a very low RID is presumed. No side effects or anomalies in infants were reported (n < 100). 44

### Dimethyl fumarate

#### *Preconception*

This medication should be avoided in patients with a desire for pregnancy and without the use of effective contraceptive methods. 45

#### *Pregnancy*

Although there is no association between adverse outcomes and pregnancies exposed to dimethyl fumarate in recent studies, 46 47 48 there is insufficient data to confirm its safety. Therefore, it is advised to stop dimethyl fumarate upon confirmation of pregnancy.

#### *Breastfeeding*

In a case report involving 2 women, dimethyl fumarate showed a RID between 0.019 and 0.007%, with a low molecular weight (130 Da). However, transfer may be reduced due to its short half-life. Therefore, breastfeeding in women using dimethyl fumarate is not recommended until more data are available. 6 49

### Teriflunomide

#### *Preconception*

This medication should not be used by pregnant women or those who may become pregnant during treatment due to its potential teratogenic effect (pregnancy category X). In cases of desire for pregnancy, it is recommended to discontinue the medication 24 months before conception or undergo accelerated elimination as follows:

- Administer 8 g of cholestyramine 3 times a day for 11 days, or 4 g of cholestyramine 3 times a day if the 8 g dose is not well tolerated.
- Alternative: Administer 50 g of activated charcoal powder every 12 hours for 11 days.

After using either accelerated elimination procedure, two separate verification tests with a 14-day interval are required, with a waiting period of 1 1/2 months between the first occurrence of a plasma concentration below 0.02 mg/L and fertilization.

Cholestyramine and activated charcoal may influence the absorption of estrogens and progestogens, potentially affecting the reliability of oral contraceptives during the elimination procedure with cholestyramine or activated charcoal. The use of an alternative contraceptive methods is recommended.

For men using teriflunomide, the risk of embryofetal toxicity is considered low, but despite this evidence, attention should be paid to possible negative fetal effects, and decision should be made on a case-by-case basis. 50

Post-marketing pharmacovigilance and recent studies do not suggest malformations or pathological changes in neonates. 51 52

#### *Pregnancy*

In case of confirmed pregnancy, discontinue the medication and undergo the accelerated elimination procedure as previously outlined. Referral to high-risk prenatal care is recommended.

Although these guidelines are used in clinical practice, as the medication demonstrated teratogenic effects in rats, recent studies do not show negative outcomes in humans. 51 One hypothesis is that there is a higher affinity for the molecule's target in rats. Therefore, further studies on the drug's kinetics and its potential teratogenicity in humans are necessary, and women exposed to teriflunomide during pregnancy should be monitored, especially with morphological ultrasound.

#### *Breastfeeding*

Due to the potential presence in breast milk (low molecular weight) and its long half-life, as well as the lack of published data on the use of the medication during breastfeeding, it should be avoided in breastfeeding mothers. 25 52

### Fingolimod

#### *Preconception*

Studies have shown an increased rate of congenital anomalies during pregnancy in humans with the use of fingolimod. Therefore, it is recommended to discontinue the medication at least two months before conception. It is important to consider the risk of inflammatory activity rebound upon discontinuation of the medication. 53 It is advisable to carefully evaluate medication change in patients with family planning < 2 years.

#### *Pregnancy*

In case of unplanned pregnancy, it is advised to stop fingolimod and switch to natalizumab during the first and second trimesters to prevent rebound activity. A study by Pauliat et al. 54 assessed congenital anomalies in fetuses exposed to fingolimod and found no statistically significant difference compared with the control group using interferon β; the limited sample (63 patients with fingolimod and 62 with interferon) does not rule out the possibility of increased risk of malformations, making its use not advisable during pregnancy. In this regard, women exposed to fingolimod during pregnancy should be monitored, especially with morphological ultrasound.

#### *Breastfeeding*

Detected in animal milk studies but lacking human breast milk data, fingolimod is estimated to be detectable due to its low molecular weight (307 Da) and long half-life. Due to the lack of data, breastfeeding while using the medication is not considered safe. 6 55

### Cladribine

#### *Preconception*

Potential risks cannot be ruled out; therefore, when attempting conception, the last dose should have been administered at least 6 months before, both in women and men. 56 It may interfere with spermatogenesis due to its effect on DNA synthesis; hence, men using it should be advised to use contraceptive methods for 6 months after the last dose of the treatment cycle. 57 58

#### *Pregnancy*

Cladribine use cannot be considered safe during pregnancy. 56

#### *Breastfeeding*

The calculated RID after 1 hour of use was 3.06% and undetectable after 48 hours. As a precaution, it is suggested that breastfeeding be suspended for 1 week after a dose of cladribine, 58 until more robust data are available.

### Natalizumab

#### *Preconception*

Portaccio et al. 59 and Ebrahimi et al. 60 found an increased number of spontaneous abortions, and Friend et al. 61 reported a higher rate of congenital malformations, although without a specific pattern. These findings were not consistent with those of the studies by Andersen et al., 46 in 2023, and Kapoor et al., 62 in 2018, and the subanalysis of the previous data did not show a significant difference compared with the general population. Thus, natalizumab can be maintained or switched to a depletion medication such as anti-CD20 or cladribine in the preconceptional period, depending on the patient's clinical characteristics. 6

#### *Pregnancy*

Studies have not demonstrated a higher risk of fetal malformations in patients using natalizumab compared with the general population, making it possible to use it in the pregnancy period. However, it is advised to use an extended protocol (1 infusion every 6–8 weeks) until the 30th to 34th week of pregnancy to reduce fetal exposure to the medication. Resume usage shortly after 1 to 2 weeks postpartum due to risk of rebound and relapses. 6 63 64

Pediatricians should be advised to collect a complete blood count, lactate dehydrogenase, and bilirubin from newborns exposed to the medication during pregnancy (especially at the end of the second trimester and during the third trimester) due to the risk of anemia and thrombocytopenia. If present, monitor these changes, which have shown spontaneous reversal in 4 months without the need for interventions. 65

#### *Breastfeeding*

Natalizumab has a low concentration in breast milk, with an average RID of 0.04% and a peak concentration between 1 and 8 days after infusion, according to the study by Proschmann et al. 66 The amount present in breast milk will likely be digested in the baby's gastrointestinal tract. 67 Therefore, there is no need for a time gap between infusion and breastfeeding, and its use is permitted during this period, provided it is a shared decision with the patient.

### Ocrelizumab

#### *Preconception*

Ocrelizumab is not associated with negative pregnancy outcomes, but it is recommended that patients wait for 1 to 3 months from the last dose before attempting pregnancy. Given the half-life (26 days) and the onset of passage through the placental barrier around 17 to 22 weeks of gestation, it can be administered close to the gestational period. 6 68 69 Assessing anti-CD19 levels may be useful during the attempt to conceive to avoid new infusions during this period, reducing the possibility of fetal exposure while ensuring the drug's efficacy for the patient.

#### *Pregnancy*

Recent studies have shown good disease control during pregnancy with preconceptional (up to 6 months before conception) or postpartum (up to 1 month after) use of ocrelizumab, which is a positive indication for using this medication in family planning for these patients. 70 71 It signals good disease control during gestation without the use of medications during this period. Infusions should be avoided during pregnancy; one possibility for monitoring would be confirming B cell levels during pregnancy. If the medication is used during pregnancy, attention should be paid to the risk of neonatal B cell depletion. It is recommended that CD19+ B cell levels are checked in the neonate exposed to the medication before proceeding with live virus vaccination. 72

#### *Breastfeeding*

Ocrelizumab has a low concentration in breast milk, with an average RID of 0.03% and a peak concentration between 1 and 7 days after infusion, becoming undetectable in 90 days. No changes were found in breastfed infants regarding B cell levels, infections, and growth and development after 1 year of follow-up. 6 72 When resuming a DMT within the first 2 weeks postpartum, it is important to consider modifying premedications for the first infusion so that prolactin levels are not affected; for instance, diphenhydramine can cause sedation and irritability in breastfed infants, and decrease milk supply after large/frequent doses. 73

### Ofatumumab

#### *Preconception*

There is no safety data available during pregnancy. It is recommended to discontinue the use of this medication before conception or as soon as pregnancy is confirmed.

#### *Pregnancy*

Data on the use of ofatumumab during pregnancy is limited due to the recent introduction of the medication, so evidence will accrue over the next few years. Recent studies with small samples have not reported negative outcomes, 74 but more research is needed. Infusions should be avoided during pregnancy; one possibility for monitoring would be confirming B cell levels during pregnancy. All live virus vaccines should be avoided in the mother and only administered to the neonate after confirming B cell levels. 75

#### *Breastfeeding*

Breastfeeding is allowed during the use of ofatumumab, with no need for a time interval between the dose and breastfeeding. 76

### Alemtuzumab

#### *Preconception*

Women of reproductive age should use effective contraceptive measures when receiving an alemtuzumab treatment cycle and for 4 months after the last infusion of each treatment cycle, as the potential risk of spontaneous abortions cannot be ruled out during or near the medication infusion.

#### *Pregnancy*

It is not recommended during pregnancy. Additional precautions should be taken:

- The use of alemtuzumab is linked to an increased incidence of other autoimmune diseases, such as immune thrombocytopenic purpura (1%) and autoimmune thyroid diseases (40%). The obstetrician should be informed about these potential diseases after treatment.
- Regarding thyroid diseases, monthly thyroid function tests should be requested during pregnancy due to the potential negative effects on both the mother and the fetus (low birth weight, preterm birth, preeclampsia, and long-term neurocognitive impairment).
- Considering the potential development of thyroid diseases in the mother after medication infusion, there may be a transfer of anti-thyroid antibodies to the fetus during pregnancy, causing Graves disease in the baby, even in euthyroid patients. This fact should be communicated to the pediatrician.

#### *Breastfeeding*

Alemtuzumab is not recommended during breastfeeding. Based on existing knowledge about other monoclonal antibodies, transmission to breast milk is likely minimal. As the medication has a long-lasting effect on the immune system, treatment during breastfeeding is rarely necessary. Breastfeeding should be discontinued during each treatment cycle and for 4 months after the last infusion of each treatment cycle. 77 78 79

## USING OR NOT USING DMTs

Each patient should have access to information about the pros and cons of using or not using DMTs during pregnancy. Therefore, the decision to maintain, suspend, or switch medications should be made with the patient, taking into account their preferences. 6

## MULTIDISCIPLINARY CARE

It is important to establish active communication with the obstetrician and pediatrician during the family planning period, clarifying important points about the disease and treatment, avoiding unnecessary iatrogenic interventions, and aligning the care plan.

## SUMMARY OF FAMILY PLANNING AMONG MS PATIENTS

**Figure 1:**
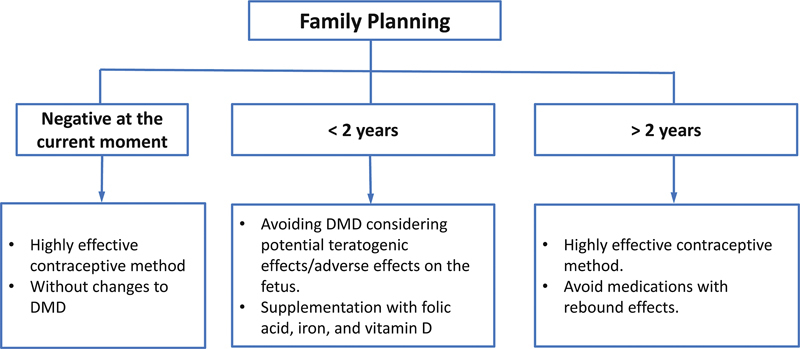
Family planning and multiple sclerosis.

**Figure 2:**
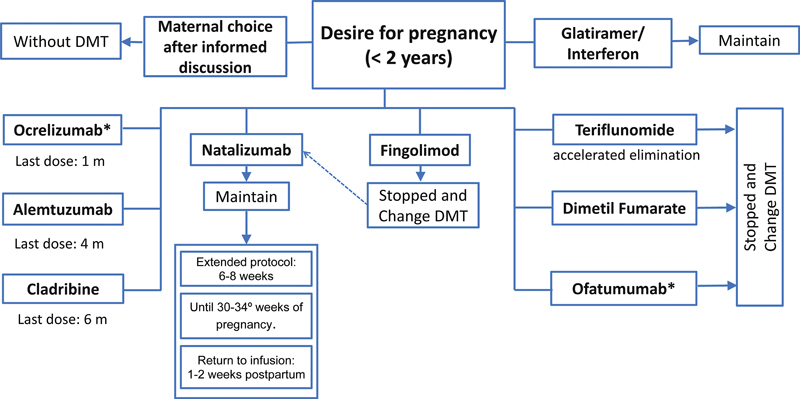
Note: *In the case of ofatumumab and ocrelizumab, one possibility for monitoring would be confirming B cell levels. Disease modifying therapies in family planning.

**Figure 3:**
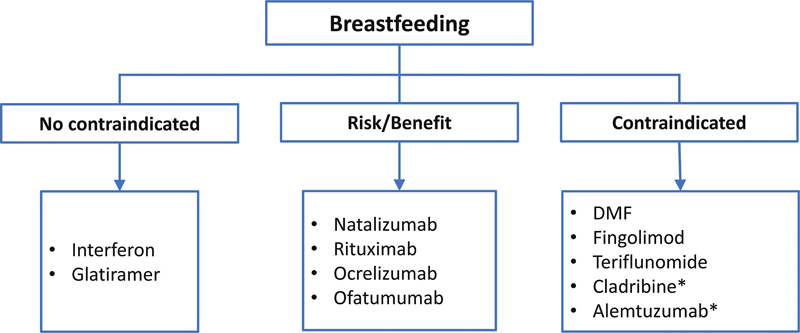
Note: *Consider breastfeeding after 7 to 10 days following the administration of Cladribine and after 4 months following the administration of Alemtuzumab. Disease modifying therapies in breastfeeding.

- It is essential to discuss family planning from the first consultation and at each subsequent appointment ( Figure 1 **)** . It is important not to delay the initiation of DMT, as we currently have safe and approved medications for use during preconception, pregnancy, and breastfeeding, which are important in preventing disability progression. This will be crucial for their ability to take care of their future family.
- Involvement of a multidisciplinary team throughout the process, including a neurologist, nurse, psychologist, physiotherapist, nutritionist, and social worker.
- To evaluate disease-modifying medication according to each patient's family planning, considering disease activity and safety for use during pregnancy and breastfeeding ( Figures 2 and 3 );
- To clarify any uncertainties to facilitate collaborative, shared decision-making;
- To ensure ongoing communication of treatment protocols and considerations related to MS to the Obstetrics and Neonatology teams;
- Staying up to date with the current guidelines and new information about the safety and long-term cohorts of both old and new therapies is necessary to improve family planning in MS.
